# Sonochemical synthesis of chalcone-functionalized SnS and SnS_2_ with improved sonocatalytic activity

**DOI:** 10.1038/s41598-026-41124-y

**Published:** 2026-05-06

**Authors:** Grzegorz Matyszczak, Konrad Głuc, Tomasz Płociński, Cezariusz Jastrzębski, Szymon Jastrzębski, Dorota Moszczyńska, Krzysztof Krawczyk

**Affiliations:** 1 Department of Chemical Technology, Faculty of Chemistry, Warsaw University of Technology, Noakowski street 3, 00-664 Warsaw, Poland; 2 Faculty of Materials Science and Engineering, Warsaw University of Technology, Wołoska street 141A, 02-507 Warsaw, Poland; 3 Faculty of Physics, Warsaw University of Technology, Koszykowa street 75, 00-662 Warsaw, Poland; 4 Faculty of Mechatronics, Warsaw University of Technology, Św. Andrzeja Boboli street 8, 02-525 Warsaw, Poland

**Keywords:** Tin(IV) sulphide, Tin(II) sulphide, Nanomaterials, Sonochemistry, Sonocatalysis, Claisen-Schmidt condensation, Chalcone, Chemistry, Materials science, Nanoscience and technology

## Abstract

Herein we present a novel method of functionalization of sonochemically obtained SnS and SnS_2_. For that purpose, a yellow-orange synthetic dye, chalcone 1,5-bis-(4-dimethylamino-phenyl)-penta-1,4-dien-3-one, was obtained via ultrasound-assisted synthesis based on the Claisen-Schmidt condensation. Then it was applied in the sonochemical synthesis of SnS and SnS_2_ microparticles using two distinct polar protic solvents – methanol and ethanol. In each of syntheses (regardless the addition of chalcone) nanocrystalline SnS_2_ phase was identified. In two cases (starting from SnCl_2_ and using methanol as solvent regardless the addition of chalcone) also more crystalline SnS phase was observed. SEM images showed that the addition of chalcone to the reaction mixture significantly affects the morphology of product, for example microparticles with near ideal spherical shape or SnS/SnS_2_ heterostructure were observed. Tauc investigations revealed the band gap widening due to the quantum confinement effect. The estimated band gap values were in the range of 1.94–4.05 eV. The addition of chalcone generally increased the band gap value. All prepared materials were tested in terms of their sonocatalytic activity towards degradation of model organic azo-dye and it was found that the chalcone-modified tin sulphide obtained from SnCl_2_ in methanol, containing SnS/SnS_2_ heterostructure, allowed for significant improvement of degradation (64%) in comparison with the unmodified sample (3%).

**Supplementary Information:**

The online version contains supplementary material available at 10.1038/s41598-026-41124-y.

## Introduction

The application of ultrasound for performing of chemical reactions started nearly one hundred years ago with the experimental discoveries of Prof. Wood and Mr Loomis^1,2^. Theoretical investigations are even older as in 1917 Lord Rayleigh reported his considerations of pressure generated during a collapse of spherical cavity inside liquid medium^3^. Nowadays it is proven that such a collapse of so-called cavitation bubbles underlies the sonochemical effect^4,5^. Ultrasound, while passing through a liquid medium, generates fluctuations of pressure that affects the solubility of gases in medium. Under certain conditions the solubility of gases, originally dissolved in the liquid medium, lowers and cavitation bubbles are formed. After several alternating cycles of rarefaction and shrinking, caused by fluctuations of pressure, the formed bubble reaches it’s maximum size (called resonant bubble radius) and collapses generating extreme conditions in terms of pressure and temperature (orders of magnitude 10^3^ atm and 10^4^ K, respectively)^4,5^.

Currently, application of ultrasound in chemistry is very wide and spans from organic to inorganic chemistry^4–9^. It is due to the many practical advantages that emerges from such approach. For example, ultrasound-assisted Claisen-Schmidt condensation reaction may be conducted much faster and in milder conditions than the classical one^10,11^. Another example of sonochemical phenomena is the synthesis of inorganic materials in which the application of ultrasound allows for avoiding of usage of toxic, health-risky solvents and high temperatures, in contrast to traditional methods of synthesis of nanomaterials such as the solvothermal or the hot injection methods^12^. Sonochemical synthesis of nanomaterials initially utilized metal complexes such as carbonyls but then moved to other reagents as inorganic salts^13^. The preparation of nanomaterials using ultrasound has two aspects. The first, chemical, aspect consists of the so-called primary sonochemistry (relying on reactions of gas-phase chemicals inside acoustic bubbles) and the so-called secondary sonochemistry (relying on reactions of solution-phase reagents outside the bubbles with radicals formed inside bubbles)^13^. The second, physical, aspect depends on physical effects resulting from acoustic cavitation and which include high-speed jets and shock waves, which may physically change particles of nanomaterials^13^.

Among many sonochemically obtained inorganic materials tin sulphides may be distinguished. The two main tin sulphides, that are intensively investigated, are SnS and SnS_2_. The first one is a p-type semiconductor with energy band gap of approximately 1.3 eV^14^. On the other hand, the second one is a n-type semiconductor with energy band gap of approximately 2.4 eV^15^. Both compounds also differ soundly in crystal structure, as SnS crystallizes in cubic or orthorombic crystal systems while SnS_2_ adopts typically hexagonal crystal structure^16,17^. Moreover, mixed tin sulphides are known, such as Sn_2_S_3_ and Sn_3_S_4_, which in fact are mixtures of SnS and SnS_2_ (1:1 for Sn_2_S_3_ and 2:1 for Sn_3_S_4_ respectively)^18–20^. Theoretical investigations of novel mixed tin sulphides, so far unknown experimentally, such as Sn_3_S_5_, Sn_4_S_5_, Sn_4_S_7_ etc., were also performed^21^. This shows that studies of tin sulphides are still very active. Some of the most recent (that is, reported since year 2024) applications of SnS include exosome detection (SnS quantum dots incorporated into mesoporous SiO_2_), solar cells (Mg-doped thin films of SnS), photocatalysis (SnS in the form of quantum dots or heterostructure), supercapacitors (SnS/multi-walled carbon nanotubes heterostructure) while those of SnS_2_ include photovoltaics (blue phosphorene/SnS_2_ heterostructure), photodetectors (SnS_2_/Te heterostructure), photocatalysis (SnS_2_ quantum dots), anode materials (reduced graphene oxide/SnS_2_ heterostructure), and supercapacitors (SnS_2_ nanoflakes)^22–29^.

Sonochemical synthesis of tin sulphides was demonstrated many times. Various studies showed that the conditions of synthesis (reagents, solvents, sonication time, and other) soundly influence the properties (such as phase purity, crystallinity, morphology, optical band gap, among others) of obtained products^30–36^. For example, a sonochemical synthesis of quantum dots of SnS and SnS_2_ from aqueous solutions was performed by Matyszczak and cooperators^25^. Quantum dots of SnS_2_ were sonochemically synthesized also using acetone as a solvent, however with much smaller yield than in the case of using aqueous solutions^25,37^. On the other hand, the synthesis of SnS quantum dots using acetone as solvent wasn’t successful^37^. This highlights the importance of choice of appropriate experimental conditions of sonochemical synthesis. Furthermore, addition of organic additives was many times applied for modification of properties (such as band gap) of nanomaterials, including sonochemically derived nano-SnS_2_ which was modified with commercial dye Phenol Red what improved it’s photocatalytic activity in the degradation of model azo-dye^38–42^.

The present study concerns the sonochemical synthesis of micropowders of SnS and SnS_2_ using novel solvent, methanol, and under the presence of artificial dye, chalcone 1,5-bis-(4-dimethylamino-phenyl)-penta-1,4-dien-3-one. According to the literature, SnS was modified with dodecylamine, oleic acid, trioctylphosphine, and hexamethyldisilazane, while SnS_2_ was modified with dye Phenol Red^38,43–45^. Thus, it is the first time when chalcone-type dye was used for that purpose. Products of syntheses are characterized by following techniques: X-ray powder diffraction (PXRD), Raman spectroscopy, Fourier transform infrared spectroscopy (FT-IR), scanning electron microscopy (SEM) coupled with energy dispersive spectroscopy (EDS), the Tauc method, and ^1^H-NMR and ^13^C-NMR spectroscopic techniques. The properties of obtained tin sulphides are determined and discussed. Synthesized materials were utilized as sonocatalysts in the process of removal of model azo-dye (Metanil Yellow) from aqueous solutions, and their activity is compared and correlated according to their key properties (morphology, phase composition, optical band gap).

The main novelty of the presented study is the utilization of artificial chalcone dye which is a novel type of organic ligand in the context of functionalization of materials. Such utilization led also to unexpected results such as the formation of SnS/SnS_2_ heterostructure. Second, marginal, novelty is related to the utilization of methanol as a solvent in the sonochemical synthesis of tin sulphides as this solvent wasn’t used so far.

## Materials and methods

### Materials and reagents

All chemicals used in this study were of analytical grade (producer: POCH). In the sonochemical syntheses following reagents were used: SnCl_4_·5H_2_O or SnCl_2_·2H_2_O and thioacetamide (TAA). Methanol or ethanol (pure for analysis) were used as a solvent during sonochemical syntheses and ethanol (denatured) was used during the purification of prepared suspensions.

### Sonochemical synthesis of chalcone (artificial dye)

**Fig. 1:**
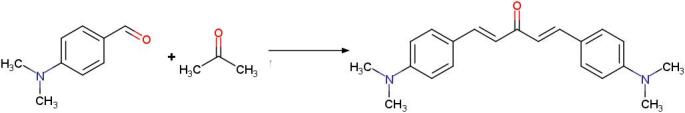
Schematic presentation of ultrasound-assisted synthesis of 1,5-bis-(4-dimethylamino-phenyl)-penta-1,4-dien-3-one chalcone via Claisen-Schmidt condensation.

As a artificial dye chalcone 1,5-bis-(4-dimethylamino-phenyl)-penta-1,4-dien-3-one was used due to the presence of large system of conjugated double C=C bonds, intensive yellow-orange colour in the visible region, and the presence of amino groups possibly allowing modification of surface of desired materials. Chalcone was obtained in ultrasound-assisted Claisen-Schmidt condensation reaction (see Fig. 1). The procedure of sonochemical synthesis of this compound was as follows: a weighed portions of 4-(dimethylamino)benzaldehyde (0.406 g) and catalyst (0.087 g, NaOH) were placed in a conical flask of 50 mL volume; then a certain volume (0.11 mL) of acetone was measured and all reagents were stirred in a 5 mL of solvent (methanol) to dissolution. Resulting reaction mixture was sonicated using ultrasonic bath (model PS-60 A) with a nominal power of 360 W. After sonication for 30 min the flask was opened and the solvent was allowed to freely evaporize yielding orange powder. The powder was purified by crystallization from 50 mL of methanol. The resulting powder was filtered under vacuum and washed with distilled water several times. The percentage yield of product was 22%. The ^1^H-NMR and ^13^C-NMR analyses confirmed the synthesis of desired compound (see Figures S1–S3 in the supplemental material).

### Procedure for sonochemical syntheses and purification of tin sulphides

The ultrasound-assisted syntheses were performed in conical flasks of 50 ml volume. As a source of ultrasound an ultrasonic bath (PS 10 A) generating wave of 40 kHz frequency was used. It’s nominal power was 60 W. The calorimetric method was used to determine acoustic power yielding value 27.9 W/L. The procedures for syntheses and purification of obtained powders were adopted, with slight modifications, from previous investigation performed by Matyszczak and cooperators^25^. First, 20 mg of synthesized chalcone (1,5-bis-(4-dimethylamino-phenyl)-penta-1,4-dien-3-one) were placed in 10 mL of corresponding solvent and stirred for 10 min to dissolution. Then certain amount of corresponding tin chloride was placed in conical flask and stirred for another 10 min. Next, 376 mg of thioacetamide were put into the conical flask and then another 10 mL of solvent was added. Finally, the reaction mixture was stirred again for another 10 min. Table 1 summarizes the amounts of reagents.

**Table 1:** Summary of information on performed sonochemical syntheses of tin sulphides.

Number of reaction	Amount of SnCl_2_·2H_2_O [mg]	Amount of SnCl_4_·5H_2_O [mg]	Amount of thioacetamide [mg]	Solvent	Desired product	Addition of chalcone
1	452	–	376	MeOH	SnS	Yes
2	452	–	376	EtOH	SnS	Yes
3	452	–	376	MeOH	SnS	No
4	452	–	376	EtOH	SnS	No
5	–	701	376	MeOH	SnS_2_	Yes
6	–	701	376	EtOH	SnS_2_	Yes
7	–	701	376	MeOH	SnS_2_	No
8	–	701	376	EtOH	SnS_2_	No

The duration of sonication was 120 min. After that and before the purification the open conical flasks with reaction mixtures were hold under laboratory hood for period of 10 min. This allowed the removal of toxic gases possibly generated during the sonochemical reaction.

The procedure of powder purification was also adopted from Matyszczak et al. investigation^25^.

The main rationale behind choice of solvents was the very good solubility of chalcone in alcohols. Additionally, there were no reports so far on the sonochemical syntheses of tin sulphides in methanol. Quantum dots of SnS and SnS_2_ were obtained sonochemically using water and, in the case of SnS2, acetone as a solvent^25,37^. Utilization of ethanol as a solvent in the sonochemical synthesis led to micro- and nanoparticles of SnS_2_, but not in the form of quantum dots^36^. Utilization of ethylenediamine as a solvent in the sonochemical synthesis allowed for synthesis of both SnS and SnS_2_ in the form of nanomaterials^36^. Therefore, it is interesting to check what results will bring the utilization of methanol as a solvent, as it wasn’t used so far in that role.

### Determination of sonochemical efficiency and percentage yield of the synthesis process

The sonochemical efficencies and percentage yields of the sonochemical syntheses of tin sulphides were determined in the same manner as in Matyszczak et al. investigation^25^.

### UV-Vis spectrophotometry (Tauc method)

The UV-Vis spectra of obtained ethanol suspensions of products of syntheses were collected using UV1600 spectrophotometer (AOE Instruments) and then were used to perform analyses based on the Tauc method.

### FT-IR investigation

For the purpose of collecting of infrared spectra a NICOLET 6700 FT-IR spectrometer was used. Spectra were measured for samples formed into pellets from dried investigated materials and KBr.

### Raman spectroscopy investigation

A Renishaw InVia Reflex in a back-scattering configuration was used to conduct Raman measurements. Laser beams with wavelengths of 1064 nm from an Nd YAG laser, 633 nm from a HeNe laser, and 514 nm from an argon ion laser were used as excitation beams. A 50x objective was used to excite and collect the scattered radiation. A 630 g/mm diffraction grating was used for 1064 nm excitation, and an 1800 g/mm grating was used for 633 and 514 nm excitations. Laser power was selected appropriately to avoid thermal changes in the sample.

### SEM investigations

The Scanning Electron Microscopy SEM investigations were performed in the same manner as in Matyszczak et al. investigation^25^.

### Powder X-ray diffraction

The material’s structure was studied using X-ray diffraction (XRD, D8 ADVANCE XRD, Bruker, Billerica, MA, USA). The measurement parameters were set as follows: CuKα radiation with a wavelength of λ = 0.154 nm, a voltage of 40 kV, and a current of 40 mA. Diffractograms were obtained within a 2θ range spanning 2–80°, with a step size of 0.02 and time 10 s.

### Sonocatalytic experiments

**Fig. 2:**
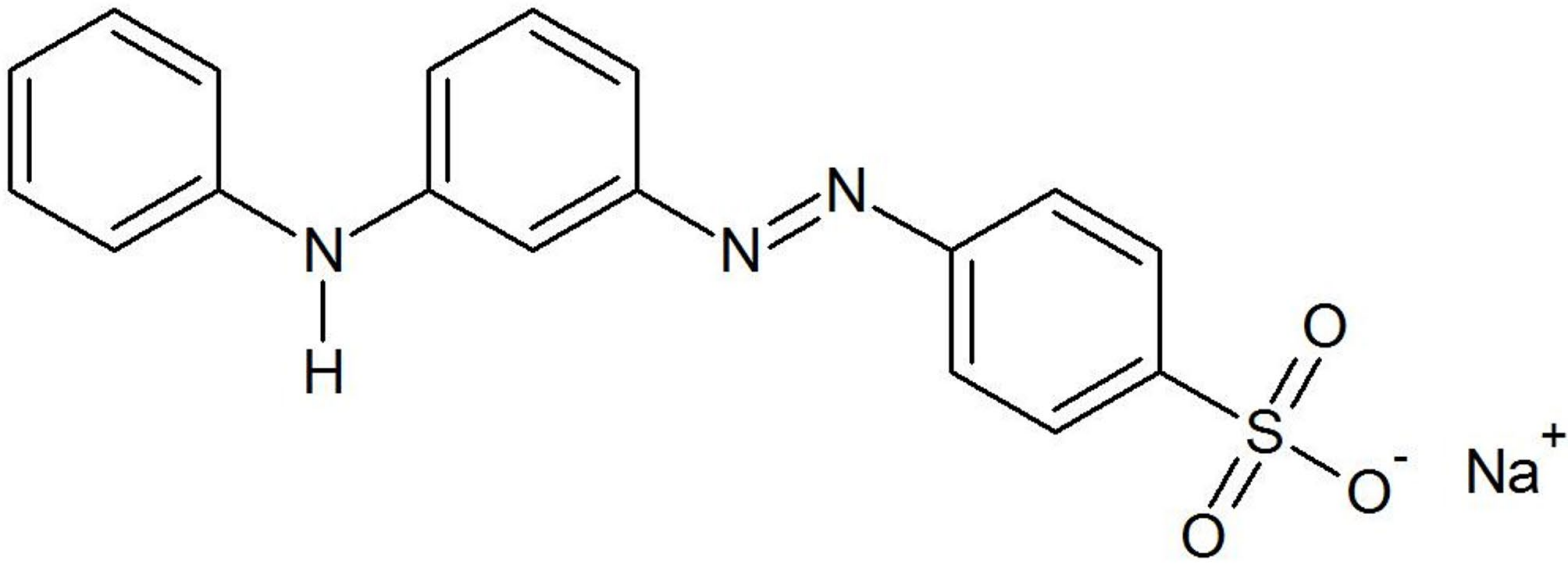
Structural formula of azo-dye Metanil Yellow.

For sonocatalytic degradation experiments a model azo-dye Metanil Yellow was chosen. It’s molecular structure is presented in Fig. 2. This organic compound is being used, despite being forbidden, in food industry in India as adulterant in turmeric powders^46,47^. However, it’s toxicity is evidenced by carcinogenic and mutagenic actions of azo-dyes against human^48–50^.

The procedure for sonocatalytic experiments was fully adopted from Matyszczak et al. investigation with slight modifications^25^.

## Results and discussion

### Investigation of syntheses efficiency

Products of syntheses formed uniform suspensions of brown or grey colours which aren’t characteristic for desired compounds (SnS – black, SnS_2_ – orange). However, it is noteworthy that the supernatants after syntheses with the addition of artificial chalcone were clear, instead of intensive yellow colour characteristic for chalcone solutions in both methanol and ethanol, suggesting that the dye was consumed during syntheses either due to its chemical transformation (e.g. possible degradation) and/or due to its incorporation into synthesized powders (hypothetically, by linking to the surface of prepared materials or by forming separate solid phase). The yield of sonochemical syntheses of tin sulphides varied with the solvent used (see Table 2). Generally, syntheses conducted in methanol were more efficient than those performed in ethanol. For both targeted compounds the greatest yield was obtained using methanol as a solvent – for SnS the percentage yield was 91.39% while for SnS_2_ it was 73.77%. For comparison, syntheses performed in ethanol resulted in percentage yields of 75.50% for SnS and 34.43% for SnS_2_, respectively. The addition of chalcone also significantly affected the yield of sonochemical synthesis (see Table 2). Such addition allowed for further increase in the percentage yields and sonochemical efficiences. For example, in the case of synthesis of SnS in methanol, the percentage yield increased from 75.17 to 91.39% with the addition of chalcone, while in the case of synthesis of SnS in ethanol the change was from 40.40% to 75.50%. In the case of synthesis of SnS_2_ in methanol the percentage yield increased from 63.93 to 73.77% with the addition of chalcone, while in the case of synthesis performed in ethanol the increase was from 7.65 to 34.43%. The positive effect of chalcone addition was more pronounced in the case of syntheses performed in ethanol. It is noteworthy that values of sonochemical efficiencies for syntheses in methanol with the addition of chalcone are only slightly lower than those for syntheses performed previously in water (12.98 mg/W)^18^.

**Table 2:** Percentage yields and sonochemical efficiencies of performed syntheses.

Number of reaction	Theoretical yield [mg]	Actual yield [mg]	Percentage yield [%]	Sonochemical efficiency [mg/W]	Chalcone addition
1	302	276	91.39	9.89	Yes
2	302	228	75.50	8.17	Yes
3	302	227	75.17	8.14	No
4	302	122	40.40	4.37	No
5	366	270	73.77	9.68	Yes
6	366	126	34.43	4.52	Yes
7	366	234	63.93	8.39	No
8	366	28	7.65	1.00	No

### X-ray powder diffraction investigations

**Fig. 3:**
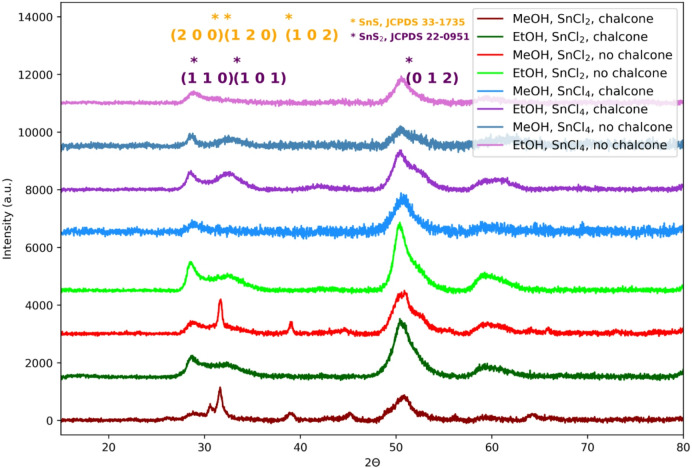
Powder X-ray diffractograms of obtained samples.

X-ray powder diffraction investigations reveal the poor crystallinity of prepared samples (see Fig. 3). However, due to the absence of wide peak in the region of low angles and, at the same time, significant widening of existing peaks it may be stated that the prepared samples are nanocrystalline. Poor quality of obtained diffractograms should cause awareness while making conclusions basing on them. Among all prepared samples evidences for SnS_2_ phase may be seen, taking into account previously reported results of SnS_2_ samples prepared in similar way^25,36^. In two samples (obtained starting from SnCl_2_ using methanol as a solvent) additional peaks, of distinct character, may be seen and they apparently correspond to the SnS phase. These peaks are soundly narrower than the other ones. Peaks at 28.5º, 33º and 51º correspond to the SnS_2_ phase, while peaks at 31º, 32º and 38.4º correspond to the SnS phase. However, due to the uncertain results of XRD investigation, other analytical techniques must be used to characterize the samples and elucidate the phase composition.

### FT-IR investigations

**Fig. 4:**
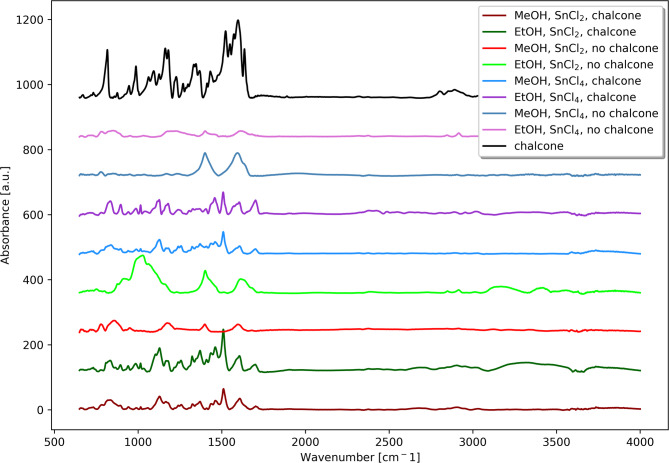
FT-IR spectra of synthesized samples and chalcone 1,5-bis-(4-dimethylamino-phenyl)-penta-1,4-dien-3-one.

The Fourier transform infrared spectroscopy investigation additionally confirms the presence of tin sulphides in unmodified samples. The corresponding FT-IR spectra are presented in Fig. 4. They show the occurrence of trace amounts of solvents that were used in syntheses and purification (C_2_H_5_OH) what is evidenced by peaks corresponding to C-H and C-C bonds, and –OH groups (bands at 1400, 1600 and 3000–3500 cm^− 1^, respectively). The bands related to Sn-S bond (range of 1000–1300 cm^− 1^) are especially pronounced in sample obtained from SnCl_2_ in ethanol^29,30^. Powders prepared with the addition of chalcone 1,5-bis-(4-dimethylamino-phenyl)-penta-1,4-dien-3-one have much reacher spectra than those unmodified, and are identical. This fact suggests that the dye is present in the same form in all modified samples. However, this form is distinct from the original dye as indicated by the comparison with FT-IR spectrum of standalone chalcone. The FT-IR spectrum of chalcone exhibits signals mostly in the fingerprint region (< 1500 cm^− 1^). The same may be seen in samples modified with chalcone, however signals are different what indicates that the chalcone underwent chemical transformations.

### Raman spectroscopy investigations

**Fig. 5:**
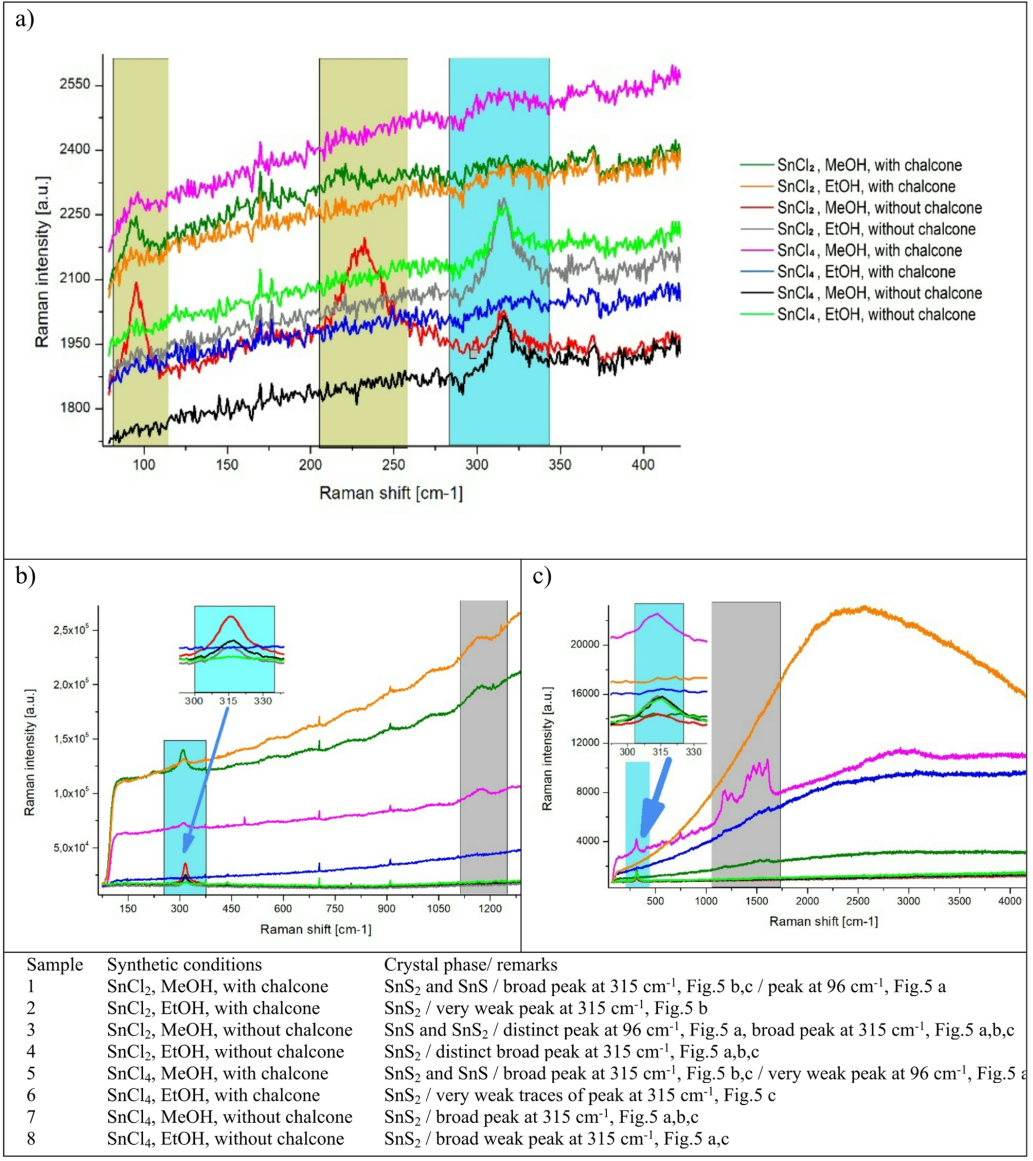
Raman spectra for samples from 8 different growth processes for different laser lines: (**a**) 1064 nm, (**b**) 633 nm, (**c**) 514 nm. Coloured areas: yellow-green, light blue, and grey, correspond to characteristic Raman peaks for the SnS, SnS_2_ crystalline phases, and organic additives used in the fabrication process, respectively. Additionally, at the bottom section, a summary table of the identified crystalline phases from Raman spectra.

Raman measurements were performed to assess the crystalline quality of the obtained samples, their phase homogeneity, inclusions of other phases and residual reaction components. SnS or SnS_2_ nanograins were expected in the fabrication process. SnS_2_ has two characteristic modes in Raman scattering with E_g_ and A_g_ symmetry, wherein the A_g_ peak located at about 315 cm^− 1^ has a relatively high intensity and can be used as an indicator of the trigonal crystalline phase of SnS_2_ with a CdI_2_-type structure and P-3m1 space group symmetry (No. 164)^51^. In the case of poor crystalline quality of SnS samples or SnS nanograins, the Raman scattering efficiency is very low, therefore the excitation wavelength of 1064 nm was used to obtain resonant enhancement of the Raman spectrum. Thanks to this, the SnS phase was observed in some samples (Fig. 5). SnS has many active modes in Raman scattering, such as: A_g_ (96 cm^− 1^), B_3g_ (164 cm^− 1^), A_g_ (192 cm^− 1^) and A_g_ (220 cm^− 1^)^52^. In resonant scattering with an unpolarized beam, the peak at approximately 96 cm^− 1^ has the highest intensity, and its presence indicates the SnS structure. Additionally, in better quality samples, the A_g_ mode can be observed at approximately 225 cm^− 1^. Other modes have relatively low intensity. Additionally, the occurrence of tin oxides was taken into account, where SnO with a tetragonal structure (P4/nmm space group) has a strong A_1g_ symmetry peak at 211 cm^−1^^53^.

Figure 5 shows typical Raman spectra obtained with different excitation lines.

Based on the yellow-green region in Fig. 5a, it can be concluded that the best crystalline SnS phase was obtained in process no. 3 (sample 3). In samples obtained in this process, a Raman signal from the SnS_2_ phase was also observed. Raman studies confirm the presence of varying quality of the SnS_2_ phase in all samples. A characteristic peak for this phase at approximately 315 cm^− 1^ is visible in each Raman spectrum; the light blue region in Figs. 5a, b, and c. The SnS_2_ samples with the best crystalline quality were obtained in processes 4 and 7 (the peak at 315 cm^− 1^ is relatively narrow and distinct). In the case of the samples obtained in process no. 1, the Raman spectrum indicates the presence of both the SnS and SnS_2_ phases. In processes no. 5 and 8, the low-intensity peak at 315 cm^− 1^ in the Raman spectrum indicates the poor quality of the SnS_2_ nanograins.

Samples obtained in process no. 6 indicate a very poor quality SnS_2_ crystal structure, while in the case of samples obtained in process no. 2, the signal from the SnS_2_ phase was covered with strong luminescence. In processes 1 and 2, and especially in process no. 5, the presence of organic components was so noticeable that not only luminescence but also Raman spectra of these substances were observed (grey area in Fig. 5b,c). Luminescence for chalcone has its maximum at approximately 560 nm. It is important to emphasize the consistency of the results obtained by the Raman and XRD methods. For samples no. 1 and 3, XRD studies recorded clear peaks for the SnS structure, and the resonance Raman spectra clearly showed the presence of this phase, Fig. 5a. Raman studies showed that in the processes in which chalcone was used (Fig. 5c), tin sulphide nanograins were decorated with chalcone molecules.

### Scanning electron microscopy investigations

**Fig. 6:**
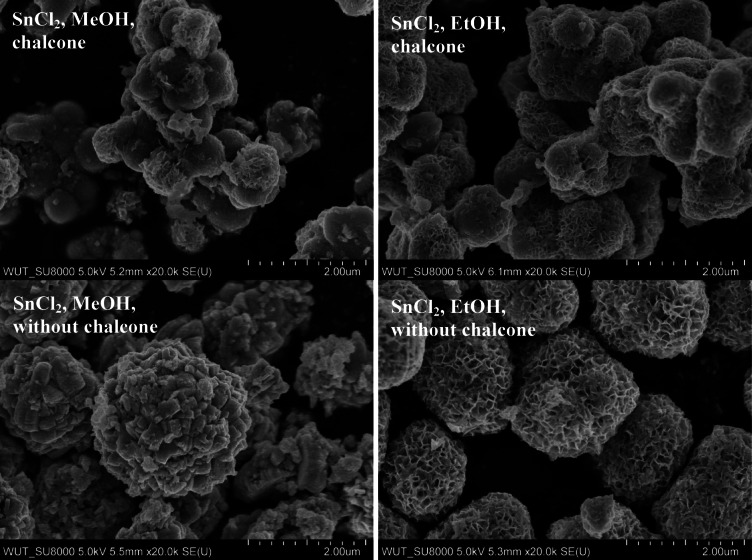

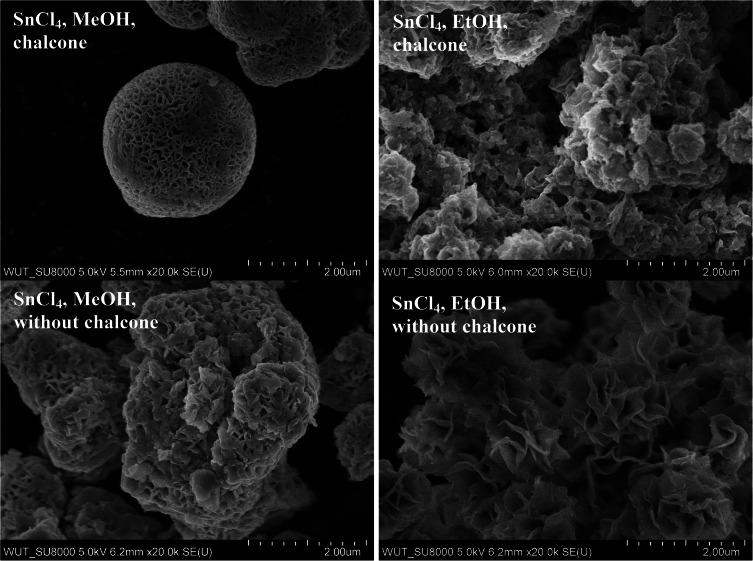
SEM images of sonochemically synthesized tin sulphides under various studied conditions (greater magnitudes).

**Fig. 7:**
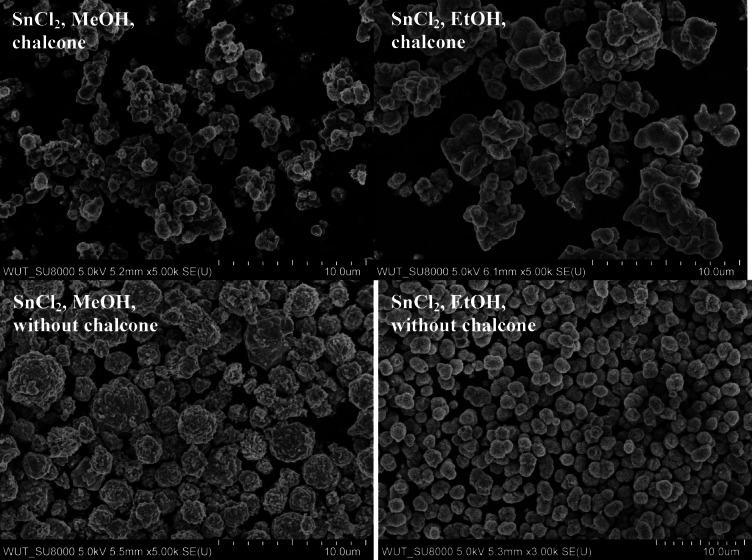

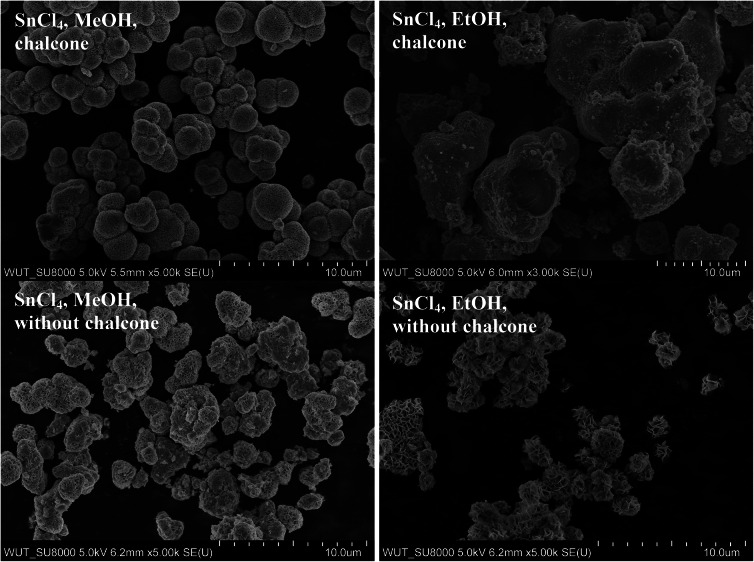
SEM images of sonochemically synthesized tin sulphides under various studied conditions (lower magnitudes).

Scanning electron microscopy observations were used to study the morphology of synthesized tin sulphides. Figure 6 presents images with greater magnitude while Fig. 7 presents images with lower magnitude. The morphology varied significantly with the synthesis conditions (solvent and addition of chalcone). However, in all cases of synthetic conditions microparticles were observed in resulting samples. Such a result is in qualitative accordance with an approximate equation that links the ultrasound frequency and resonance bubble radius reached in the moment of it’s collapse^54^:

$$\:f\cdot\:R\approx\:3$$

where: f – ultrasound frequency [Hz]. R – resonance bubble radius [m].

According to the above equation, the resonance bubble radius under applied frequency of ultrasound (which is 40 kHz in this study) is 75 micrometers. As may be seen from Figs. 6 and 7 the obtained samples contain relatively uniform microparticles of size lying in the apparent range of 1 to 5 micrometers, however their size is one magnitude lower than the calculated resonance bubble radius. This discrepancy may be caused by the approximate nature of utilized equation, however it is noteworthy that obtained results can’t be physically negated as it would be in the case when the calculated resonance bubble radius would be lesser than the size of synthesized particles.

The micropowder synthesized in ethanol and starting from SnCl_4_ tin precursor exhibited flower-like morphology typical for such synthetic conditions^36,38^. It was previously proven that such micropowder in fact consists of organized elongated nanostrains^35^. Turning the solvent to methanol while retaining the kind of tin precursor resulted in similar flower-like morphology however with apparent greater agglomeration. On the other hand, retaining both solvent (ethanol) and tin precursor (SnCl_4_) with addition of chalcone resulted in product with also similar flower-like morphology but less pronounced. Interesting result was obtained in the case of addition of chalcone, but using methanol as a solvent and SnCl_4_ as tin precursor. Powder obtained under such conditions also exhibited flower-like morphology, however it’s almost ideal spherical shape is distinctive among all obtained samples.

Syntheses starting from SnCl_2_ using ethanol as a solvent gave flower-like microparticles with denser appearance than in the case of product of syntheses starting from SnCl_4_ tin precursor. Also in this case addition of the chalcone to the reaction mixture led to the less-pronounced flower like morphology (see Fig. 6 for conditions: SnCl_2_, EtOH, chalcone). With the change of solvent from ethanol to methanol, but retaining SnCl_2_ as a tin precursor, the morphology changes drastically. A globular, agglomerated microparticle may be seen in a figure corresponding to the conditions: SnCl_2_, MeOH, without chalcone (Fig. 6). Interestingly, the addition of chalcone in the case of synthesis performed in methanol and starting from SnCl_2_ resulted in formation of two kind of particles – one with flower-like morphology and second with smooth spherical-like morphology.

The results of EDS elemental composition investigations (presented as Figures S4–S11 in supplemental material) suggest the formation of SnS_2_ compound under all investigated synthetic conditions. This is indicated by near-ideal Sn: S atomic ratio lying in the range of 0.49 to 0.59 (Table 3). However, in two cases another phase is indicated by the EDS investigation. In the case of sample prepared using SnCl_2_ as a tin source and methanol as a solvent the Sn: S atomic ratio is 1.06 what is characteristic for SnS phase. This is the only sample that didn’t exhibit flower-like morphology (Fig. 6). Taking into account the XRD and Raman spectroscopy results it may be concluded that this sample contains SnS-SnS_2_ heterostructure formed as a core-shell system, in which the core is made of SnS_2_ phase while the shell is made of SnS phase. As XRD investigations showed, sample obtained under similar conditions (SnCl_2_, methanol) but with the addition of chalcone also contained two phases, however according to corresponding SEM image they are separated with SnS showing smooth, spherical-like morphology and SnS_2_ showing it’s typical flower-like morphology. For this sample, EDS results also suggest the coexistence of two phases indicated by following Sn: S atomic ratios: 0.77 for SnS and 0.59 for SnS_2_.

**Table 3:** Results of the EDS elemental composition (at %) of tested samples.

Sample	Sn: S atomic ratio
1	0.77, 0.59
2	0.57
3	1.06
4	0.59
5	0.55
6	0.53
7	0.49
8	0.55

### Determination of energy band gap values using the Tauc method

The Tauc method was used to investigate the optical energy band gaps of synthesized materials. The analysis was narrowed to the direct allowed transitions. Results of investigation are collected in Table 4 below. The values varied soundly among samples and lied in range starting from 1.94 eV to 4.05 eV, indicating quantum confinement effect as the band gap values for bulk SnS and SnS_2_ compounds are ca. 1.3 and 2.4 eV, respectively^14,15^. This gives another evidence for the presence of nanoparticles in synthesized samples. Regarding the addition of chalcone, it generally led to the significant increase in the value of optical band gap (3.87 vs. 2.49 eV, 3.65 vs. 3.13 eV, and 4.05 vs. 1.94 eV). Only when starting from SnCl_4_ and using methanol as a solvent the addition of chalcone led to decrease of the band gap from 3.23 eV (unmodified sample) to 2.64 eV (chalcone-modified sample). The direct allowed transition was assumed because typically this kind of transition was observed for sonochemically obtained tin sulphides^25,35–38^.

**Table 4:** Values of optical band gaps for direct allowed transition for synthesized samples.

Sample	Synthetic conditions	Band gap [eV]
1	SnCl_2_, MeOH, with chalcone	3.87
2	SnCl_2_, EtOH, with chalcone	3.65
3	SnCl_2_, MeOH, without chalcone	2.49
4	SnCl_2_, EtOH, without chalcone	3.13
5	SnCl_4_, MeOH, with chalcone	2.64
6	SnCl_4_, EtOH, with chalcone	4.05
7	SnCl_4_, MeOH, without chalcone	3.23
8	SnCl_4_, EtOH, without chalcone	1.94

Corresponding Tauc plots are provided in supplementary materials (Figs. S12–S19).

### Sonocatalytic activity

**Fig. 8:**
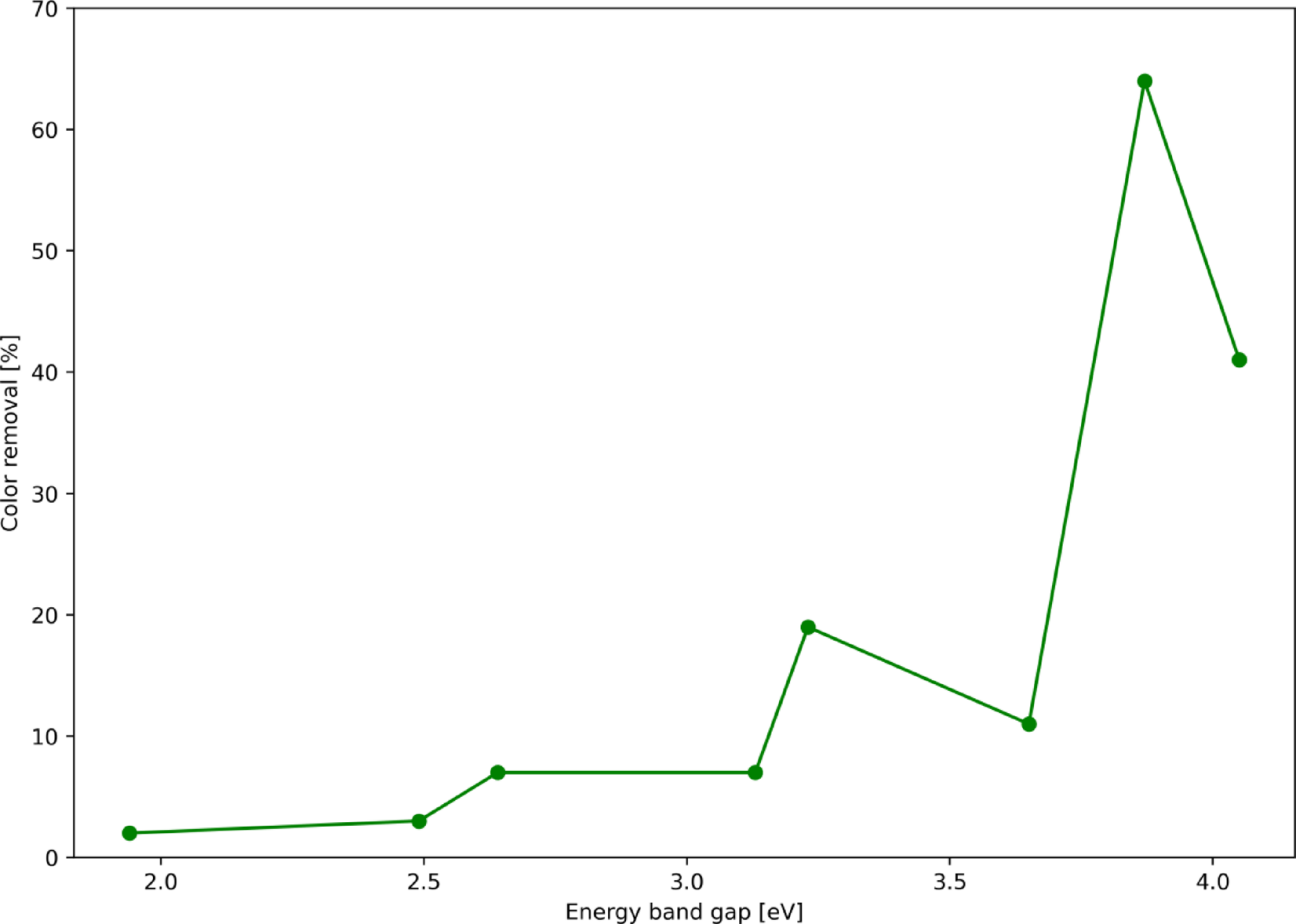
A correlation between color removal and optical energy band gap among synthesized samples of tin sulphide.

Each of synthesized samples was used as a sonocatalyst in the process of degradation of model azo-dye, Metanil Yellow. The calculated percentages of color removals are collected in Table 5. It may be seen that generally the addition of chalcone improves the sonocatalytic activity as two the best samples (64 and 41% removal, respectively) were modified with chalcone. In contrast, respective unmodified powders exhibited color removals of only 3 and 2%, respectively. However, such observation is not true in the case of SnS_2_ sample obtained starting from SnCl_4_ in methanol because in this case the color removal lowered from 19 to 7% after the modification with chalcone. In the case of SnS_2_ obtained starting from SnCl_2_ in ethanol, the modification with chalcone contributed to only slight increase in color removal, from 7 to 11%. By comparing these results with Fig. 7 it seems that the existence of more spherical and uniform particles in powder doesn’t go in pair with sonocatalytic activity. Greater color removal was observed for samples that contained less regular agglomerates. The highest sonocatalytic activity was exhibited by sample clearly containing two phases, SnS and SnS_2_, connected with each other thus forming heterostructure in which the separation of charge carriers may be enhanced. However, this is not observed in the unmodified sample no. 3 (that is, obtained starting from SnCl_2_ in methanol, without chalcone) which also contained both phases, as indicated by XRD, Raman spectroscopy, SEM and EDS investigations, and possible SnS/SnS_2_ heterostructure as well. It should be recalled that the SnS phase in this sample covers the SnS_2_ phase, thus blocking the effective separation of charge carriers – SnS_2_ simply doesn’t have contact with the reaction medium during sonocatalytic process. Taking into account the values of band gaps, apparently there is also a positive correlation between them and sonocatalytic activity. Generally, lower sonocatalytic activity was observed for samples with lower value of optical band gap (see Fig. 8). Taking into account that the sonocatalytic effect has three main underlying mechanisms: thermal excitation, photoexcitation (by photons emitted due to the sonoluminescence) and enhanced acoustic cavitation, it may be speculated that the photoexcitation mechanism plays the most important role among studied sonocatalysts^55^.

Maximum color removal of 64% obtained in this study may look moderate. However, after comparing it to values reported in literature for sonocatalytic degradation of Metanil Yellow such a value reflects relatively good efficiency. Sonocatalytic degradation of Metanil Yellow by SnS and SnS_2_ quantum dots reached maximally 85.2% and 39.3%, respectively^25^. It should be emphasized that the form of quantum dots is especially efficient in sonocatalysis due to the extreme lowering of the Gibbs free energy for formation of acoustic bubbles^55^. Even so, tin sulphide obtained from SnCl_2_ in methanol with the addition of chalcone did better than SnS_2_ quantum dots.

**Table 5:** Values of color removal in the process of sonocatalytic degradation of Metanil Yellow for all synthesized samples.

Sample	Synthetic conditions	Color removal [%]
1	SnCl_2_, MeOH, with chalcone	64
2	SnCl_2_, EtOH, with chalcone	11
3	SnCl_2_, MeOH, without chalcone	3
4	SnCl_2_, EtOH, without chalcone	7
5	SnCl_4_, MeOH, with chalcone	7
6	SnCl_4_, EtOH, with chalcone	41
7	SnCl_4_, MeOH, without chalcone	19
8	SnCl_4_, EtOH, without chalcone	2

## Conclusions

We have sonochemically synthesized various SnS_2_ samples in the form of microparticles starting from SnCl_2_ or SnCl_4_ and using two organic solvents (methanol or ethanol) with or without the addition of artificial organic dye, chalcone 1,5-bis-(4-dimethylamino-phenyl)-penta-1,4-dien-3-one. Chalcone was synthesized using ultrasound-assisted Claisen-Schmidt condensation. Two of prepared samples contained also SnS phase alongside the SnS_2_ phase. In one such case a open SnS/SnS_2_ heterostructure was obtained, while in second case such heterostructure had a core-shell form in which the shell was build of SnS. The addition of chalcone to the reaction mixture during the synthesis significantly influenced the properties of obtained products. While the morphology of obtained SnS_2_ samples was generally flower-like, it was more dense in the case of syntheses performed with the addition of chalcones. Such addition also allowed for opening of the SnS/SnS_2_ heterostructure, from a core-shell structure, as a result of synthesis. The optical band gap for direct allowed transition was generally greater for samples modified with chalcone. The same was observed for sonocatalytic activity in the process of degradation of model azo-dye Metanil Yellow. In general, powders modified with chalcone allowed for greater color removal in comparison with those unmodified. The greatest sonocatalytic activity was observed for sample containing SnS/SnS_2_ heterostructure under the modification with chalcone − 64% color removal - while similar sample, but containing only SnS_2_ phase showed only 41% color removal. This study opens new perspective in the control of properties of inorganic materials produced sonochemically as well as highlights the need of future investigations on influence of organic additives during sonochemical synthesis. The optimization of amount of organic additive and investigation of novel organic ligands for modifications of materials are two main proposed future directions related to this study.

## Supplementary Information

Below is the link to the electronic supplementary material.

Supplementary Material 1

## Data Availability

The datasets used and/or analysed during the current study are available from the corresponding author on reasonable request.

## Declarations

### Generative AI and AI-assisted technologies in the writing process

Generative AI and AI-assisted technologies were not used in the writing process.
