# Supplementary material for: Sonochemical synthesis of chalcone-functionalized SnS and SnS2 with improved sonocatalytic activity
Source: Sci Rep. 2026 May 6;16:20731. doi: 10.1038/s41598-026-41124-y (PMC13333848; doi:10.1038/s41598-026-41124-y)
Supplement: Supplementary file 1 — Supplementary Material 1 [file 41598_2026_41124_MOESM1_ESM.docx]

Supplemental information for:

Sonochemical synthesis of chalcone-functionalized SnS and SnS_2_ with improved sonocatalytic activity

Grzegorz Matyszczak^a)^*, Konrad Głuc^a)^, Tomasz Plocinski^b)^, Cezariusz Jastrzebski^c)^, Szymon Jastrzębski^d)^, Dorota Moszczyńska^b)^, Krzysztof Krawczyk^a)^

a) Department of Chemical Technology, Faculty of Chemistry, Warsaw University of Technology, Noakowski street 3, 00-664 Warsaw

b) Faculty of Materials Science and Engineering, Warsaw University of Technology, Wołoska street 141A, 02-507 Warsaw

c) Faculty of Physics, Warsaw University of Technology, Koszykowa street 75, 00-662 Warsaw

d) Faculty of Mechatronics, Warsaw University of Technology, Św. Andrzeja Boboli street 8, 02-525 Warsaw

Corresponding author: Grzegorz Matyszczak, grzegorz.matyszczak@pw.edu.pl


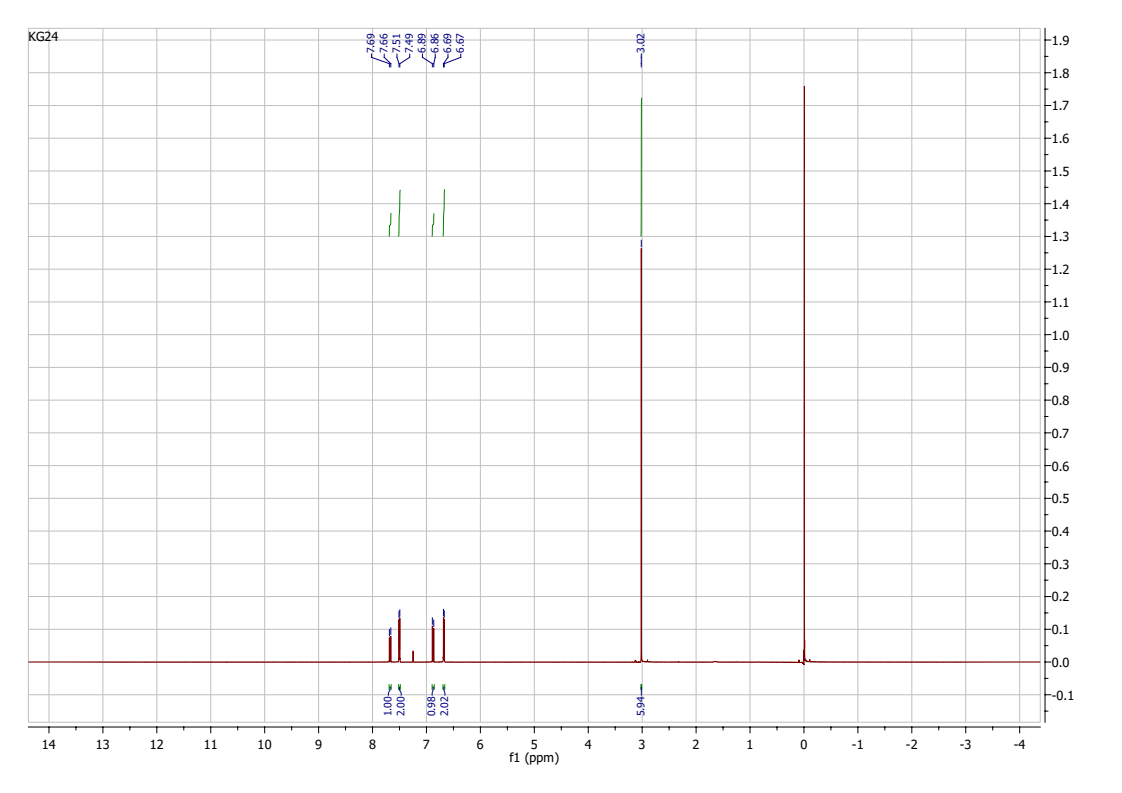


**Figure S1.** ^1^H-NMR spectrum of synthesized chalcone.


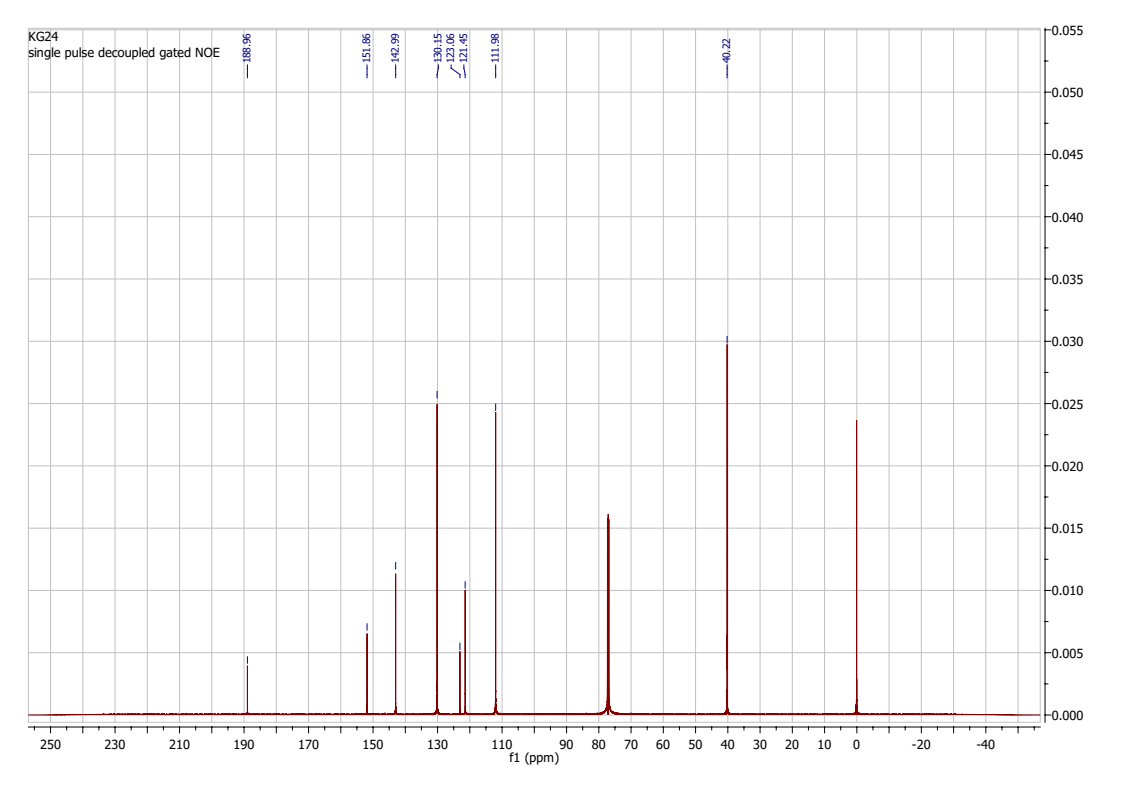


**Figure S2.** ^13^C-NMR spectrum of synthesized chalcone.


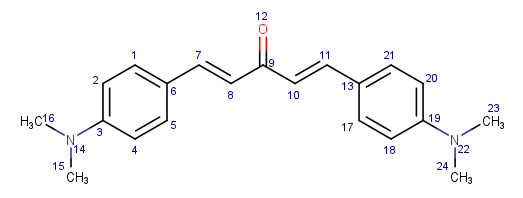


**Figure S3.** Structure of synthesized chalcone with atoms enumerated for NMR analyses.

^1^H NMR (600 MHz, CDCl_3_): δ (ppm): 7,67 (d, J = 15,7Hz, 2H, H-7 i H-11), 7,51-7,49 (m, 4H, H-1, H-5, H-17 i H-21), 6,88 (d, J = 15,7Hz, 2H, H-8 i H-10), 6,69-6,67 (m, 4H, H-2, H-4, H-18, H-20), 3,02 (s, 12H, H-15, H-16, H-23, H-24)

^13^C NMR (150 MHz, CDCl_3_): δ (ppm): 189,0 (C-9), 151,9 (C-3 i C-19), 143,0 (C-7 i C-11), 130,1 (C-1, C-5, C-17 i C-21), 123,0 (C-6 i C-13), 121,4 (C-8 i C-10), 112,0 (C-2, C-4, C-18 i C-20), 40,2 (C-15, C-16, C-23 i C-24)


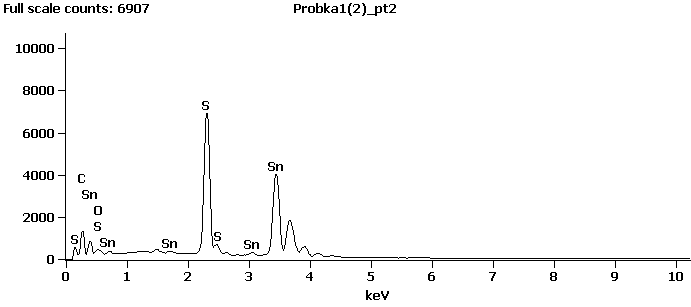


**Figure S4.** EDS spectrum for tin sulphide obtained starting from SnCl_2_ and using methanol as solvent, with the addition of chalcone.


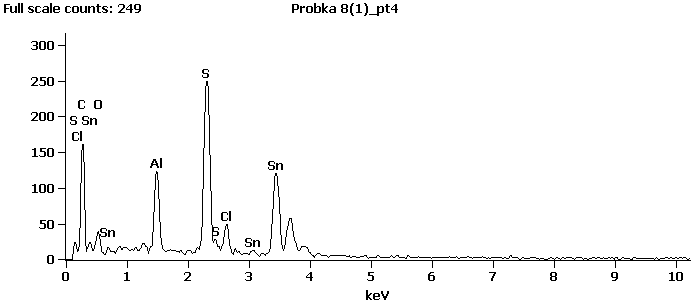


**Figure S5.** EDS spectrum for tin sulphide obtained starting from SnCl_2_ and using ethanol as solvent, with the addition of chalcone.


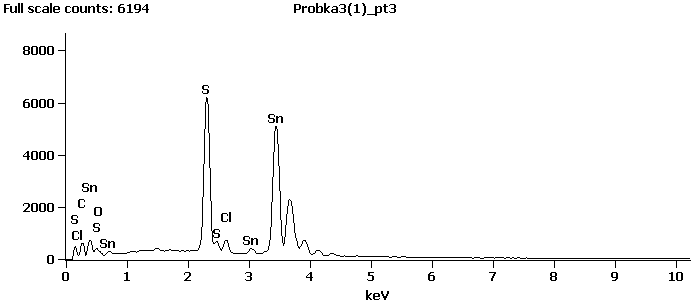


**Figure S6.** EDS spectrum for tin sulphide obtained starting from SnCl_2_ and using methanol as solvent, without the addition of chalcone.


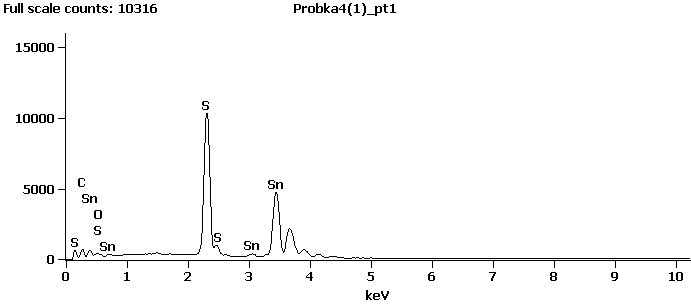


**Figure S7.** EDS spectrum for tin sulphide obtained starting from SnCl_2_ and using ethanol as solvent, without the addition of chalcone.


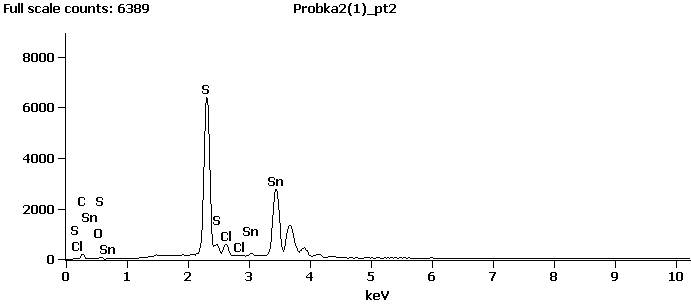


**Figure S8.** EDS spectrum for tin sulphide obtained starting from SnCl_4_ and using methanol as solvent, with the addition of chalcone.


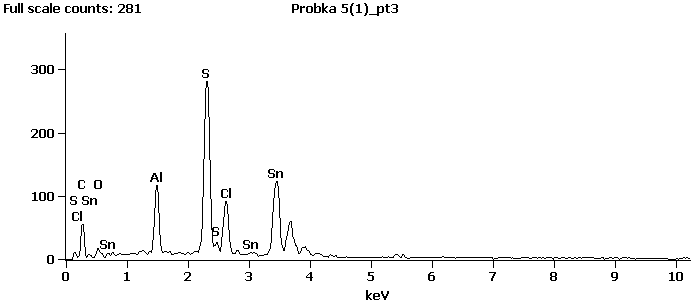


**Figure S9.** EDS spectrum for tin sulphide obtained starting from SnCl_4_ and using ethanol as solvent, with the addition of chalcone.


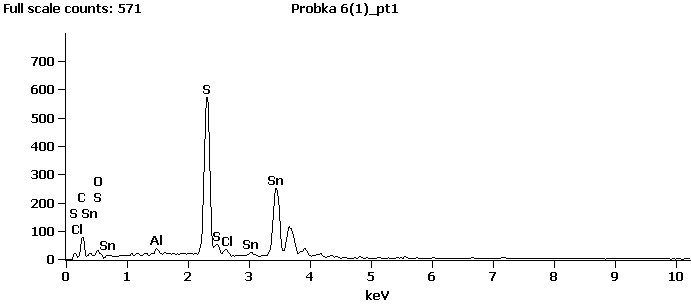


**Figure S10.** EDS spectrum for tin sulphide obtained starting from SnCl_4_ and using methanol as solvent, without the addition of chalcone.


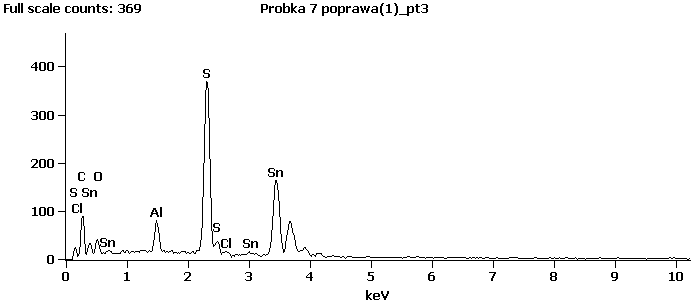


**Figure S11.** EDS spectrum for tin sulphide obtained starting from SnCl_4_ and using ethanol as solvent, without the addition of chalcone.

**Figure S12.** The Tauc plot for tin sulphide obtained from SnCl_2_ in methanol with the addition of chalcone, corresponding to the direct allowed transition.

**Figure S13.** The Tauc plot for tin sulphide obtained from SnCl_2_ in ethanol with the addition of chalcone, corresponding to the direct allowed transition.

**Figure S14.** The Tauc plot for tin sulphide obtained from SnCl_2_ in methanol without the addition of chalcone, corresponding to the direct allowed transition.

**Figure S15.** The Tauc plot for tin sulphide obtained from SnCl_2_ in ethanol without the addition of chalcone, corresponding to the direct allowed transition.

**Figure S16.** The Tauc plot for tin sulphide obtained from SnCl_4_ in methanol with the addition of chalcone, corresponding to the direct allowed transition.

**Figure S17.** The Tauc plot for tin sulphide obtained from SnCl_4_ in ethanol with the addition of chalcone, corresponding to the direct allowed transition.

**Figure S18.** The Tauc plot for tin sulphide obtained from SnCl_4_ in methanol without the addition of chalcone, corresponding to the direct allowed transition.

**Figure S19.** The Tauc plot for tin sulphide obtained from SnCl_4_ in ethanol without the addition of chalcone, corresponding to the direct allowed transition.
